# 5*d* Orbital Covalency Controls the High‐Pressure Polymorphism of BaO

**DOI:** 10.1002/chem.202501536

**Published:** 2025-08-18

**Authors:** Sophie Kogan, Anastassia N. Alexandrova, Harry W. T. Morgan

**Affiliations:** ^1^ University of California, Los Angeles Department of Chemistry and Biochemistry 607 Charles E Young Drive East Los Angeles CA 90095 USA; ^2^ Department of Chemistry University of Manchester Oxford Road Manchester M13 9PL UK

**Keywords:** ab initio calculations, bond theory, high pressure, phase transitions, *s*‐block metals

## Abstract

The influence of metal *d* orbitals on the high‐pressure structures of CaO, SrO, and BaO are investigated with DFT calculations and chemical bonding analysis. CaO and SrO undergo the *B*1‐*B*2 transition, from the rock salt structure to the caesium chloride structure, while BaO undergoes a sequence of transitions from *B*1‐*B*8, the NiAs structure, and then *B*8 − *dB*2, a distorted form of *B*2. DFT calculations of bond strengths show that the *B*8 and *dB*2 structures are stabilized relative to *B*1 and *B*2 by metal–oxygen covalency through the metal *d* orbitals. In BaO covalency outweighs electrostatics because of the large 5*d* orbitals of Ba, so the unique *B*8 and *dB*2 structures form. This marks an important expansion of the importance of *d* orbitals in group II chemistry.

## Graphical abstract

Barium oxide forms unique structures under pressure because of Ba—O bonding through the 5*d* orbitals. DFT bonding analysis shows that these bonds outweigh electrostatic interactions in BaO, but not CaO or SrO.

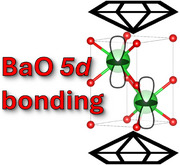

## 1 Introduction

The chemistry of barium, strontium, and calcium is dominated by ionic compounds in which the metals adopt the +2 oxidation state.^[^ ^1^ ^]^ However, recent investigations into the organometallic chemistry of these metals have found that they can interact with ligands covalently through *d* orbitals.^[^ ^2^ ^]^ *d* orbital covalency is demonstrated elegantly by the complexes M(CO)_8_, M(N_2_)_8_ (M = Ca, Sr, Ba) and M(C_6_H_6_)_3_ (M = Sr, Ba), which are more like transition metal compounds than typical alkaline earth metal compounds.^[^ ^3^, ^4^, ^5^, ^6^ ^]^ They obey the 18‐electron rule of organometallic chemistry, which requires the filling of bonding orbitals formed between the five metal *d* orbitals and the ligand orbitals. There are also iron complexes featuring metal–metal bonding to calcium, strontium, and barium through their *d* orbitals,^[^ ^7^ ^]^ and calcium complexes in which calcium bonds to ligands through the 3*d* orbitals; the oxidation state of calcium in these complexes is unclear due to M‐L bonding, but they react like Ca(I) compounds.^[^ ^8^, ^9^, ^10^ ^]^ Earlier theoretical work established a trend of increasing *d* orbital participation in molecules going down group II.^[^ ^11^, ^12^, ^13^ ^]^

These developments invite us to look for other areas of *s*‐block chemistry in which metal *d* orbitals play an important role. The metallic conductivities of the Zintl phases Ba_3_Si_4_ and Ca_5_Ge_3_ are inconsistent with the Zintl–Klemm concept, which assumes complete transfer of the cation valence electrons to the anions, and have instead been rationalized by covalent interactions between the cation *d* and anion *p* orbitals.^[^ ^14^, ^15^ ^]^ In this study, we will study simple metal oxides with density functional theory (DFT) and use high pressure to explore unusual bonding environments.

**Figure 1:**
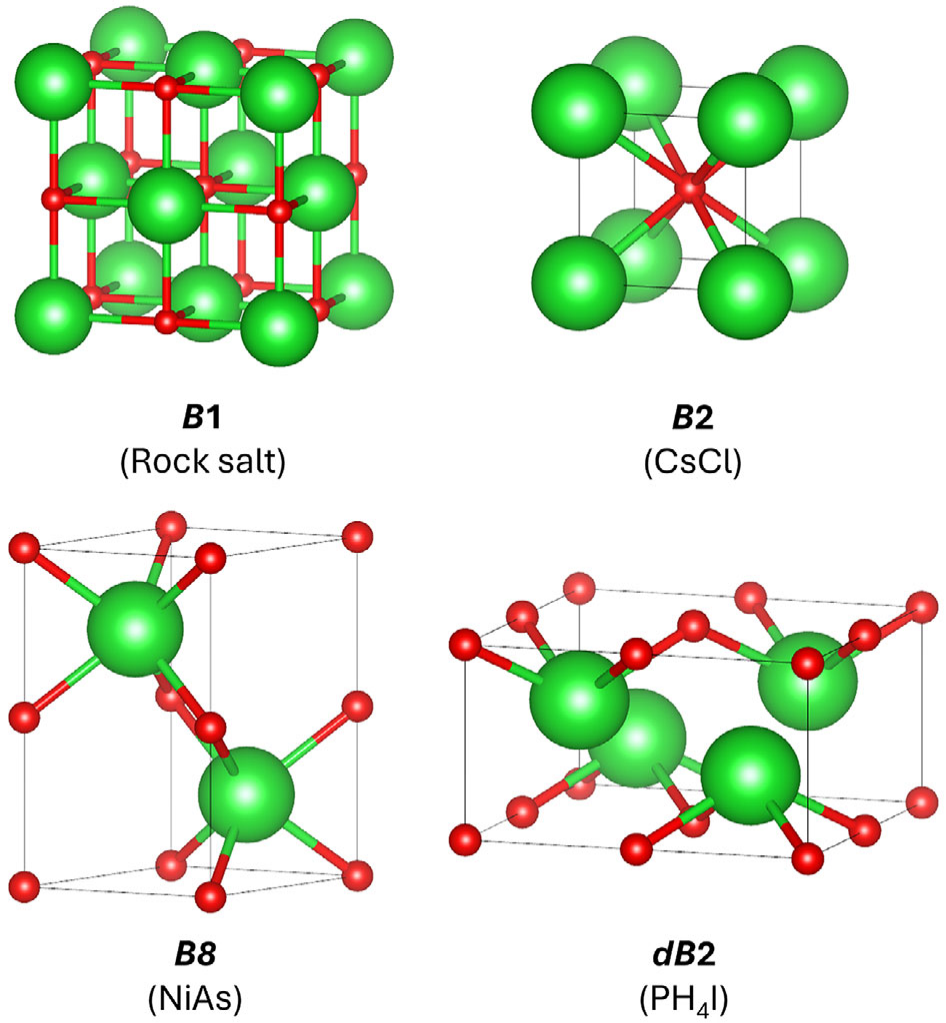
Unit cells of BaO in the *B*1, *B*2, *dB*2, and *B*8 structures.

Many ionic materials with AB stoichiometry, such as group I metal halides and group II metal oxides, adopt the rock salt (*B*1 in Strukturbericht nomenclature) structure at ambient pressure. Under high pressure, the majority of these undergo a phase transition to the CsCl (*B*2) structure.^[^ ^16^ ^]^ The *B*1‐*B*2 transition can be easily understood as a change from 6‐coordinate to 8‐coordinate geometry to reduce the unit cell volume, minimizing enthalpy under pressure at the expense of internal energy. CaO and SrO follow this pattern, with critical pressures of 64 GPa and 36 GPa, respectively.^[^ ^17^, ^18^, ^19^ ^]^ However, unlike other *s*‐block oxides and halides, BaO adopts the NiAs (*B*8) structure at 10 GPa and then a distorted CsCl structure, called *dB*2 or the PH_4_I structure, at 15 GPa.^[^ ^20^ ^]^ All four structures are shown in Figure 1. The *B*8 structure has the cation in trigonal prismatic coordination, while the oxides remain in octahedral coordination. It is typically adopted by compounds with a mixture of ionic and covalent interactions such as NiAs. In the *dB*2 structure, the metal is displaced away from the center of an O_8_ cube along the *c* direction. The thermodynamics of the transitions and the mechanical properties of the various phases have been studied extensively, but no explanation of this polymorphism has been given.^[^ ^21^, ^22^, ^23^, ^24^ ^]^

In transition metal chemistry, it is well known that 5*d* metals form stronger covalent bonds than 3*d* metals, so there may be a covalent explanation for the unique behaviour of BaO. The structural complexity of elemental barium under pressure has been explained by electron transfer between the 6*s* and 5*d* valence orbitals,^[^ ^25^ ^]^ elemental caesium also undergoes a continuous 6*s* − 5*d* transition under pressure,^[^ ^26^, ^27^ ^]^ and a predicted high‐pressure phase unique to BaB_6_ has also been attributed to covalent effects.^[^ ^28^ ^]^ In this report, we will show that covalent bonding through the 5*d* orbitals is responsible for the appearance of the *B*8 and *dB*2 phases of BaO, highlighting the importance of *d* orbital covalency in alkaline earth M^2 +^ compounds.

## 2 Methods

DFT calculations were performed with VASP version 5.4.4.^[^ ^29^, ^30^ ^]^ All calculations used the PBE functional and a plane‐wave cut‐off of 600 eV.^[^ ^31^ ^]^ The *k*‐point grid used for each phase is shown in Table 1.

**Table 1:** *k*‐point grids.

Phase	*k*‐mesh
*B*1	5‐5‐5
*B*2	7‐7‐7
*B*8	7‐7‐4
*db*2	4‐4‐8

Local‐orbital wavefunction projections were done with LOBSTER, version 5.0.0.^[^ ^32^ ^]^ The basis functions were taken from the set developed by Koga et al., with modifications that allowed us to investigate metal *d* orbital effects (see below).^[^ ^32^, ^33^, ^34^ ^]^ Other high‐energy diffuse functions (e.g., Ca 4*p*) were excluded from the projection basis in line with previous testing of LOBSTER on high‐pressure materials.^[^ ^35^ ^]^

## 3 Results and Discussion

### 3.1 Transition Pressures

**Figure 2:**
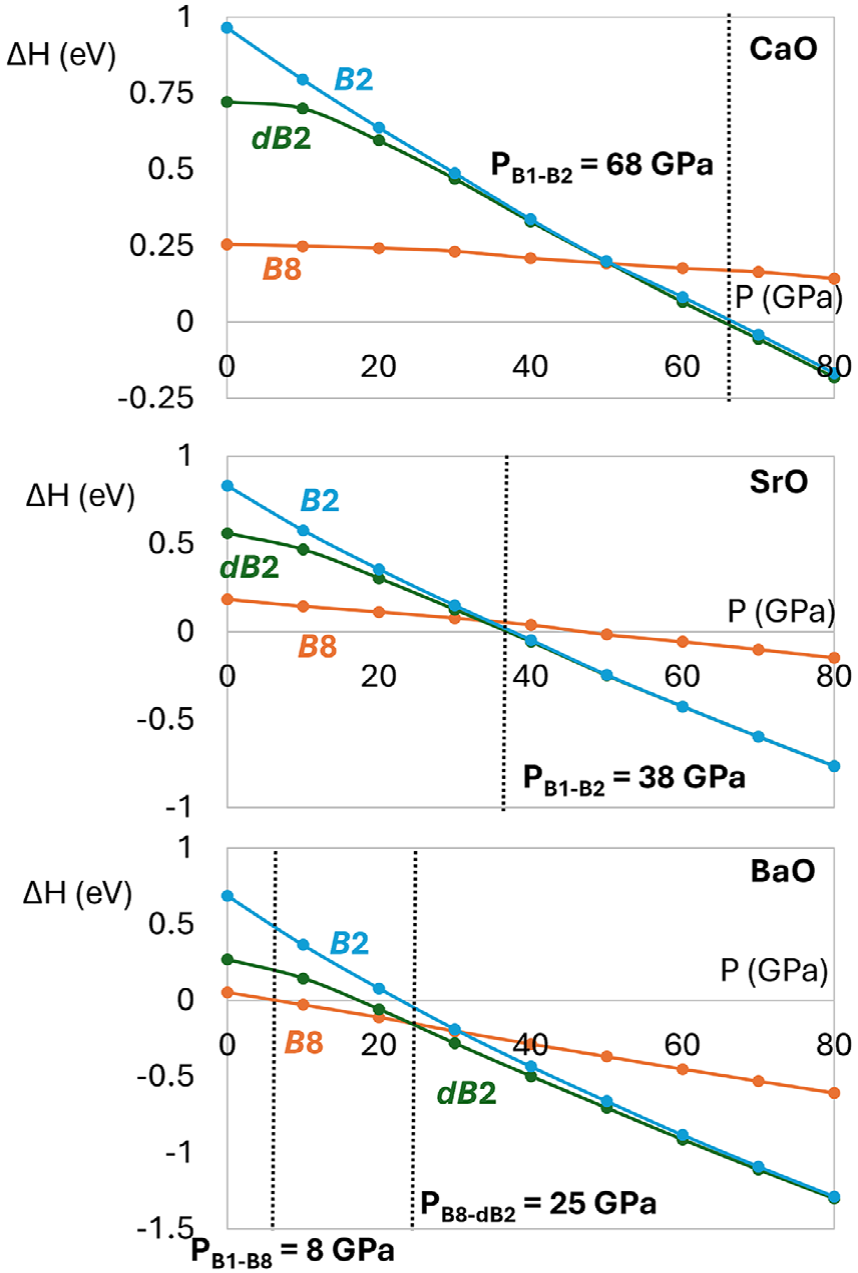
Enthalpies of the *B*8, *B*2, and *dB*2 phases relative to *B*1 of CaO, SrO, and BaO. Vertical dashed lines mark the computed transition pressures.

We optimized CaO, SrO, and BaO in the four structures *B*1, *B*2, *B*8, and *dB*2 at pressures up to 80 GPa. Figure 2 shows the enthalpies of all structures relative to *B*1 for each compound. Our results agree with previous experiments and computations.^[^ ^20^, ^21^ ^]^ At ambient pressure, all compounds adopt the *B*1 structure. According to our calculations, CaO transitions to the *B*2 structure above 68 GPa, and SrO undergoes the same transition at about 38 GPa. For CaO and SrO the *B*2 and *dB*2 points coalesce above 30 GPa because *dB*2 optimizes to the undistorted *B*2 form. We compute the critical pressure for each transition as the pressure at which the two phases have equal enthalpies. Previous work on vibrational effects on the *B*1 − *B*2 transition in CaO and SrO found that zero‐point motion has a negligible effect on the critical pressures, likely because all the atoms are heavy, and thermal effects do not significantly change the critical pressures until the temperature is higher than 500 K.^[^ ^23^ ^]^

Instead of *B*1‐*B*2, BaO undergoes a *B*1‐*B*8 transition at around 10 GPa, and a *B*8‐*dB*2 transition at around 25 GPa. The *dB*2 and *B*2 structures become more similar as the pressure increases further, but *dB*2 remains the ground state throughout the studied pressure range. The main thermodynamic driving force for these transitions is volume reduction, which substantially reduces the *PV* component of the enthalpy at high pressure. Figure S1 shows *PV* for each structure, relative to *B*1, and for each compound. The *B*8 structures have similar *PV* to *B*1 because the ions are 6‐coordinate in both cases, while the 8‐coordinate *B*2 and *dB*2 phases give much larger *PV* reductions.

### 3.2 Energetic Analysis

To understand the unique polymorphism of BaO we performed energetic analysis on all phases of CaO, SrO, and BaO at all pressures using LOBSTER.^[^ ^32^ ^]^ LOBSTER projects the delocalized electronic wavefunction onto atomic orbitals, allowing us to compute intuitive energetic contributions like atomic charges, Madelung energies, and pairwise covalent interaction energies through the Crystal Orbital Hamiltonian Population (COHP).^[^ ^36^, ^37^ ^]^ The polymorphism of Be_*x*_Mg_1 − *x*_O compounds was studied recently by similar methods.^[^ ^38^ ^]^

When doing a local orbital projection with LOBSTER it is important to choose which atomic orbitals to use in the projection basis, i.e., the atomic orbitals from which to reconstruct the wavefunction. For example, for oxygen we would expect the 2*s* and 2*p* orbitals to describe the chemistry; for a 1st row transition metal we would typically include the 3*d*, 4*s*, and 4*p* valence orbitals, and sometimes it may be important to include the semi‐core 3*s* and 3*p* states. For group II metals, the ordinary basis orbitals would be the semi‐core (*n* − 1)*s* and (*n* − 1)*p* and the valence *ns* that are empty in a M^2 +^ cation. For strontium this would be 4*s*, 4*p*, and 5*s*. This neglects the *nd* orbitals, and in fact the standard basis set libraries in LOBSTER do not contain *nd* functions for the group II metals. Since we are interested in the *d* orbitals we must construct a custom basis set that contains them. Following a procedure used to study Ba_3_Si_4_, we added *d* functions to the basis set taken from the next atom in the periodic table (i.e., Sc 3*d* was added to Ca, Y 4*d* to Sr, and La 5*d* to Ba).^[^ ^14^ ^]^

The quality of the projection is measured by the “charge spilling,” which measures the success of the relocation of electrons from the delocalized wavefunction into the atomic orbital bases. A perfect projection would have a charge spilling of 0%, and values below 3% are typically considered accurate enough for chemical analysis.^[^ ^39^ ^]^ We therefore used the charge spilling as a measure of the importance of *d* functions for a high‐quality localized wavefunction. Across all three compounds in all phases at all pressures, inclusion of *d* orbitals in the projection basis reduces the charge spilling by approximately 50%. The effect is particularly important for BaO, where the charge spilling without 5*d* orbitals is as high as 5.13%, but it is no higher than 3.35% when 5*d* orbitals are included. Increasing pressure causes the charge spilling to increase slightly, consistent with previous work.^[^ ^35^ ^]^ Full results are available in the Supporting Information.

Having done a local projection of the wavefunction we can quantify the covalent interaction energies between pairs of atoms with the Integrated Crystal Orbital Hamiltonian Population (ICOHP). We have computed the ICOHP for the nearest‐neighbour M–O pair in each structure as a function of pressure with and without metal *d* orbitals in the projection basis. Without *d* orbitals, the M–O ICOHPs are small (∼0.5 eV), with no significant differences between structures and no dependence on pressure.

**Figure 3:**
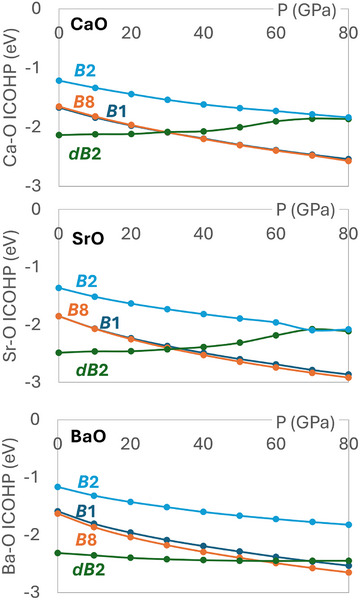
ICOHPs per bond for nearest‐neighbour M─O bonds in CaO, SrO, and BaO in all phases as a function of pressure. A more negative ICOHP indicates a more stabilizing M─O bonding interaction.

When we add *d* orbitals the M–O ICOHPs are affected dramatically. Graphs of M─O ICOHP versus pressure, for a single M─O bond, are shown in Figure 3. The four structures now have clear differences between them and the ICOHPs are pressure‐dependent. The general trend, common to all three oxides, is that *B*2 has the smallest ICOHP, *B*1 and *B*8 have similar M–O ICOHPs to each other, and that the *B*1, *B*2, and *B*8 ICOHPs all become larger as pressure increases. *dB*2 shows different behaviour: at 0 GPa it has the most negative ICOHP for all compounds, but does not become more negative with increasing pressure as the other phases do. This can be attributed to the fact that *dB*2 is strongly distorted at low pressure but the extent of the distortion decreases as pressure increases, so the M─O bonds are less strongly affected by pressure in this structure.

To understand the appearance of the *B*8 and *dB*2 phases in BaO we must look more closely at the differences between the compounds. The ICOHP difference between *B*8 and *B*1 increases down the group, implying greater covalent stabilization of *B*8 for BaO. This difference is plotted as a function of pressure in Figure S5. The same trend is not observed in the ICOHP plots without *d* basis functions, so we can conclude that the *B*8 phase of BaO is stabilized by M─O bonding through the 5*d* orbitals.

The *dB*2 ICOHP behaves differently between the compounds; in BaO it stays approximately constant as pressure increases, but in CaO and SrO it rises, implying decreasing covalent stabilization, merging with the *B*2 curve at 70‐80 GPa. In CaO and SrO, the greatest enthalpic benefit comes from reducing volume by adopting the undistorted *B*2 structure, at the expense of M–O covalent stabilization, while in BaO the distortion is maintained to minimize the electronic energy. This effect can also be seen in the difference between the M─O bond lengths in the *B*2 and *dB*2 structures, as plotted in Figure S6. Therefore, the *dB*2 phase also appears in BaO due to *d* orbital covalency.

A database of computed ICOHP values for a wide range of compounds shows that ICOHPs for interactions in the same length range as our computed M─O bonds (2.25–3.0 Å) are typically between 0 and ‐6 eV.^[^ ^39^ ^]^ Our ICOHPs, from ‐1 to ‐3 eV, therefore represent weak but significant covalent interactions, particularly given the large electronegativity difference between *s*‐block metals and oxygen, which is generally associated with weak covalent bonding and small ICOHP.

We can also consider the electrostatic contribution to the lattice enthalpy, which can be calculated by using the projected orbital populations to define atomic charges and then treating those as point charges to calculate the Madelung energy. The Madelung energies as a function of pressure are plotted in Figure S7. All three compounds show the same general trend ‐ *B*1 is most stable, *dB*2 is least stable, and *B*8 and *B*2 are intermediate. The trend is largely pressure‐independent, though *B*2 and *dB*2 are stabilized at higher pressures relative to *B*1 and *B*8. Greater electrostatic stabilization of *B*1 than *B*8 is the main reason for which the *B*8 phase is not observed for most group II oxides and group I halides. The Madelung energies become less negative as pressure increases for all phases. We believe that this reflects increasing covalency at high pressure, which reduces the computed atomic charges and therefore the magnitude of the Madelung energy decreases. Note that the *B*2 structure, adopted by all compounds in the high‐pressure limit, is not favoured energetically by either covalent or electrostatic effects. It is therefore adopted because it minimizes enthalpy by having the lowest volume at high pressure, which outweighs energetic factors.

These results have broader implications for *d* orbital‐driven chemistry of Ca, Sr, and Ba. Computational basis sets frequently exclude the valence *d* orbitals of Ca, Sr, and Ba, so *d* orbital covalent effects are easily missed. Given the growing list of group II compounds that display *d* orbital covalency it might be useful to parameterize basis sets with *d* functions. Only BaO has a sufficient covalent driving force to adopt the unusual *B*8 and *dB*2 structures, suggesting that those seeking to stabilize organometallic complexes with group II metals in low oxidation states should focus on Ba(I) rather than Ca(I). It is clear that the chemistry of the group II metals is not entirely dominated by closed‐shell ionic interactions, and interesting phenomena may be observed if we can expand the chemical space of group II *d* orbital chemistry.

## 4 Conclusions

We have investigated the high‐pressure structural phase transitions of CaO, SrO, and BaO with DFT calculations including local bonding analysis. The structures of interest are *B*1, the NaCl structure, *B*2, the CsCl structure, *B*8, the NiAs structure, and *dB*2, a distorted form of *B*2 adopted by PH_4_I. CaO and SrO undergo a single *B*1 − *B*2 transition, at 68 GPa and 38 GPa respectively, in common with many ionic oxides, sulfides, and halides. BaO undergoes a sequence of transitions from *B*1‐*B*8 (8 GPa) and then *B*8 − *dB*2 (25 GPa), with *dB*2 smoothly turning into the undistorted *B*2 structure as the pressure increases. Using ICOHP calculations to assess the metal‐oxygen bond strengths we have shown that the *B*8 and *dB*2 phases of BaO are stabilized relative to *B*1 and *B*2 by metal–oxygen covalency through the barium 5*d* orbitals. Covalency is strong enough to be structure‐directing for BaO but not CaO or SrO, in keeping with the trend of 5*d* transition metals forming the strongest covalent bonds. Electrostatics favor the ambient‐pressure *B*1 structure, while volume reduction drives the oxides to adopt the *B*2 structure in the high‐pressure limit. The unique transition sequence of BaO is therefore due to *d* orbital‐based covalency.

## Conflict of Interest

There are no conflicts of interest to declare.

## Supporting information

Supporting Information

## Data Availability

The data that support the findings of this study are available in the supplementary material of this article.
