# Supplementary material for: 5d Orbital Covalency Controls the High‐Pressure Polymorphism of BaO
Source: Chemistry. 2025 Aug 18;31(50):e202501536. doi: 10.1002/chem.202501536 (PMC12415313; doi:10.1002/chem.202501536)
Supplement: Supplementary file 1 — Supporting Information [file CHEM-31-e202501536-s001.pdf]

Supporting Information: $5d$ Orbital Covalency Controls the High-Pressure Polymorphism of BaO

Sophie Kogan,[†] Anastassia N. Alexandrova,[†] and Harry W. T. Morgan[*,†,‡]

†*Department of Chemistry and Biochemistry, University of California, Los Angeles, Los Angeles, CA 90095, USA*

‡*Department of Chemistry, University of Manchester, Oxford Road, Manchester M13 9PL, UK*

E-mail: harry.morgan@manchester.ac.uk

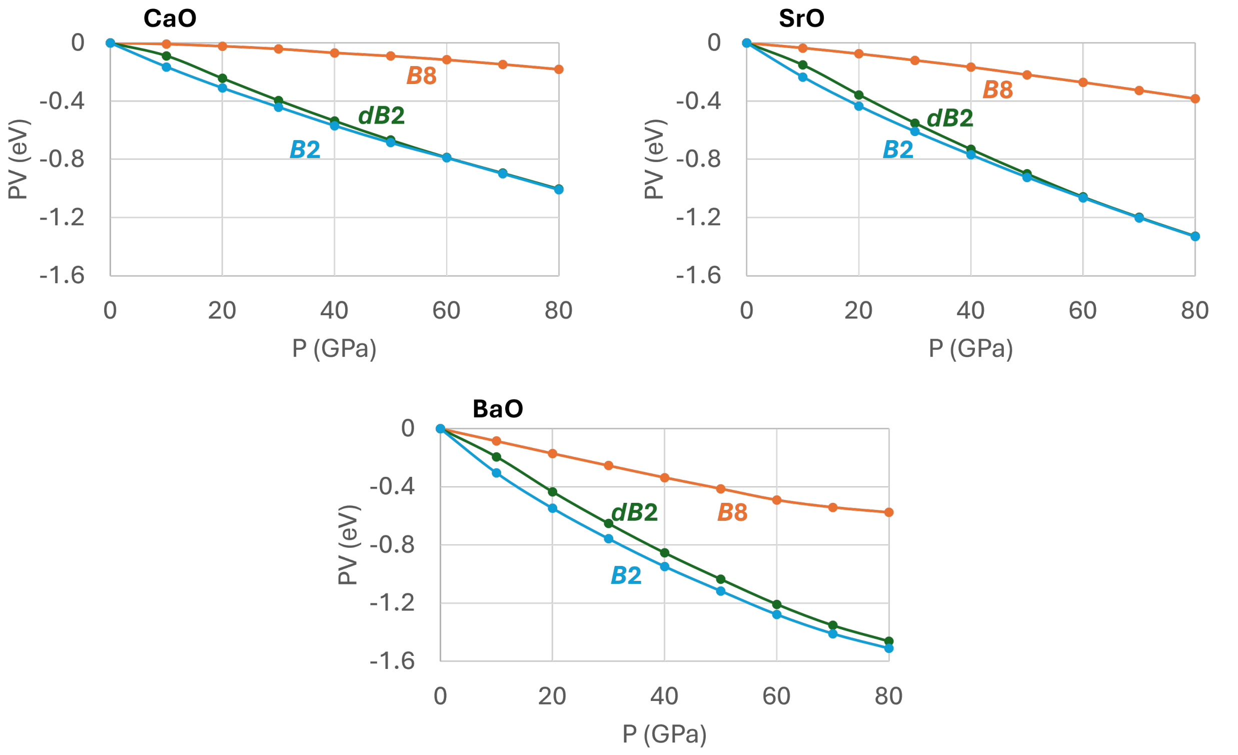

Figure S1: PV contribution to the enthalpies of the $B8$, $B2$, and $dB2$ phases relative to $B1$ of CaO, SrO, and BaO.

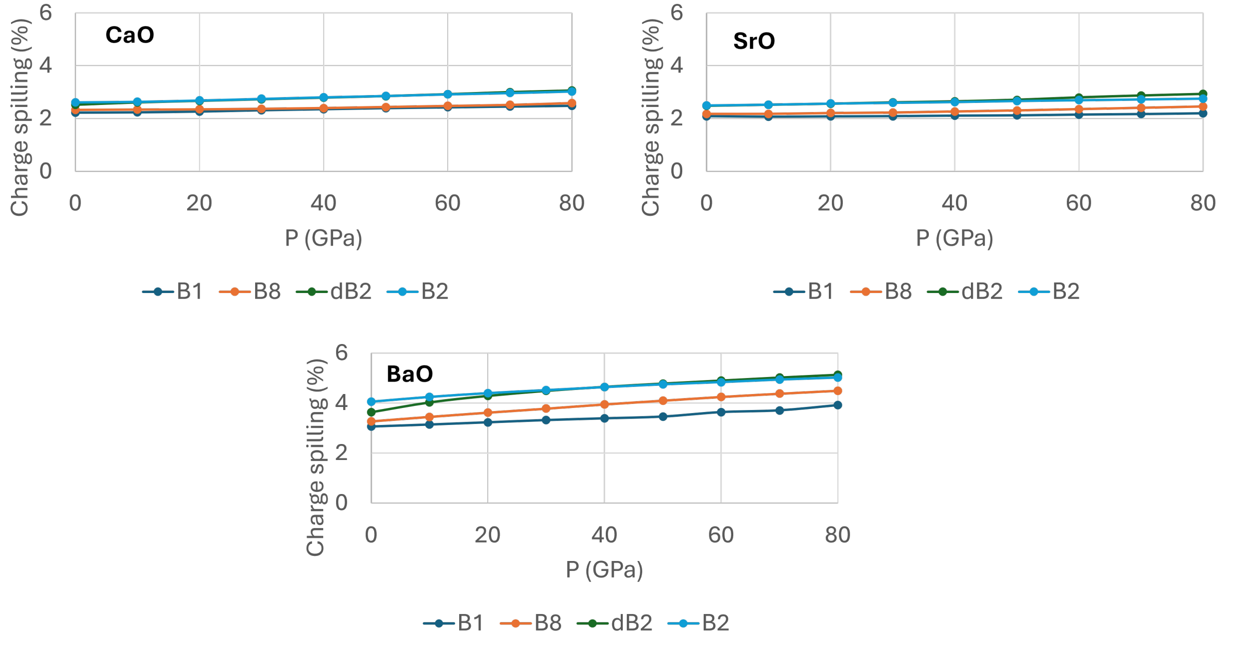

Figure S2: Charge spilling in all structures of CaO, SrO, and BaO as a function of pressure computed without d functions in the projection basis

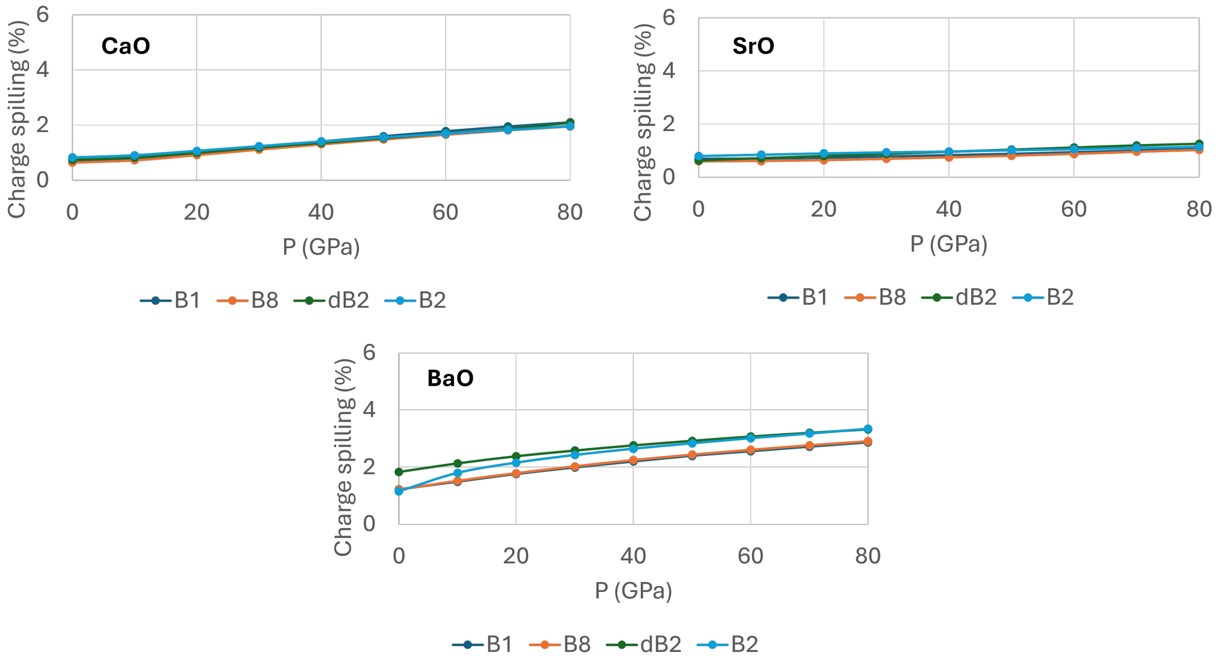

Figure S3: Charge spilling in all structures of CaO, SrO, and BaO as a function of pressure computed with d functions included in the projection basis

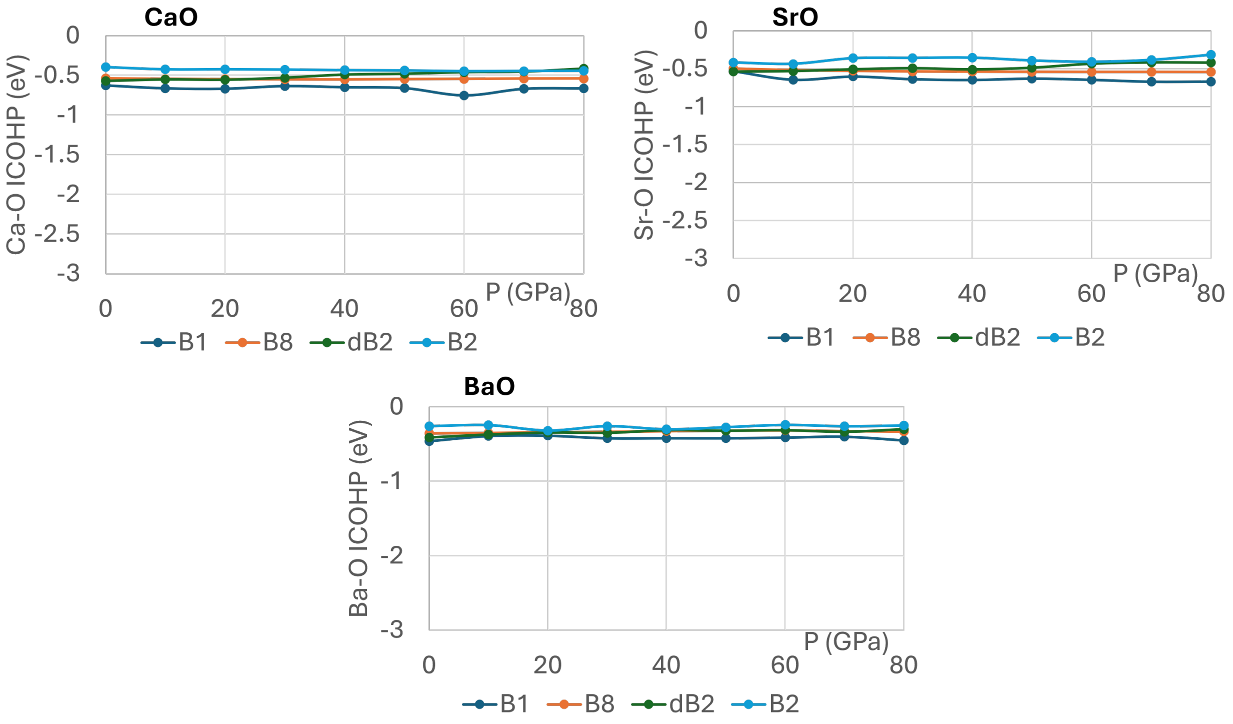

Figure S4: ICOHPs for nearest-neighbour M-O bonds in CaO, SrO, and BaO in all phases as a function of pressure without d functions in the projection basis.

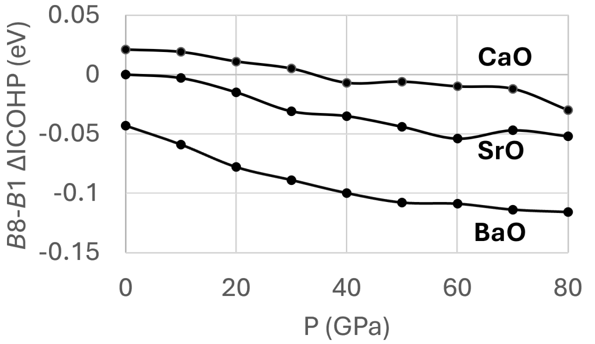

Figure S5: M-O ICOHP difference between $B8$ and $B1$ phases of CaO, SrO, and BaO as a function of pressure. A negative value indicates covalent stabilization of $B8$ relative to $B1$.

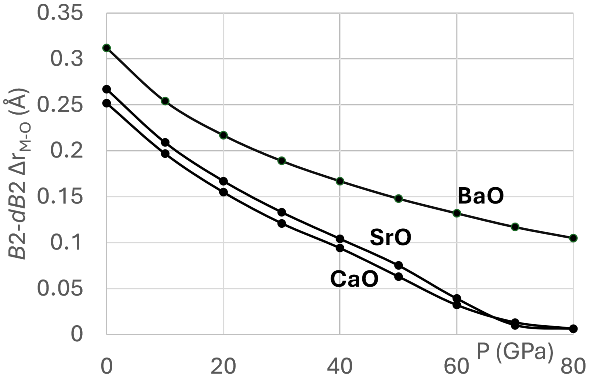

Figure S6: Difference between the nearest-neighbour M-O bond lengths in the $B2$ and $dB2$ structures for CaO, SrO, and BaO as a function of pressure.

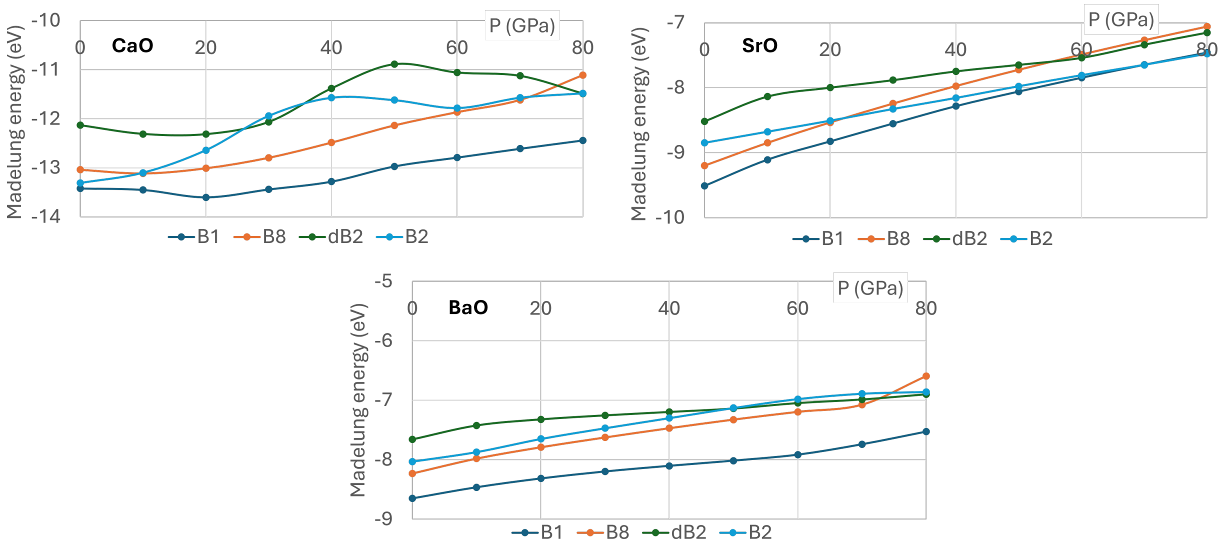

Figure S7: Madelung energies of CaO, SrO, and BaO in all phases computed using Mulliken charges with d functions included in the projection basis
